# Placebo-resistant gut bacteria: *Akkermansia muciniphila* spp. and Familial Mediterranean fever disease

**DOI:** 10.3389/fcimb.2024.1336752

**Published:** 2024-02-23

**Authors:** Elya Pepoyan, Francesco Marotta, Anahit Manvelyan, Artak Galstyan, Lena Stepanyan, Hasmik Grigoryan, Liana Grigoryan, Mikayel Mikayelyan, Marine Balayan, Natalya Harutyunyan, Susanna Mirzabekyan, Vardan Tsaturyan, Tamas Torok, Astghik Pepoyan

**Affiliations:** ^1^ Food Safety and Biotechnology Department, Scientific Research Institute of Food Science and Biotechnology, Armenian National Agrarian University, Yerevan, Armenia; ^2^ International Association for Human and Animals Health Improvement, Yerevan, Armenia; ^3^ Faculty of Military Medicine, Yerevan State Medical University, Yerevan, Armenia; ^4^ ReGenera R&D International for Aging Intervention, Milan, Italy; ^5^ Lawrence Berkeley National Laboratory, Berkeley, CA, United States

**Keywords:** placebo, male patients, microbiome, *Akkermansia muciniphila*, familial Mediterranean fever, *Enterobacteriaceae* spp., *Faecalibacterium*, *Blautia*

## Abstract

**Introduction:**

Despite numerous investigations into the impact of drugs/probiotics on the gut microbiota composition in Familial Mediterranean Fever (FMF) patients, the question as to whether there exists a significant bacterial diversity(ies) independent of the placebo effect that can be reliably considered in clinical and nutritional trials remains unresolved.

**Methods:**

This study represents the in augural analysis of the placebo’s influence on the gut microbiota of both healthy individuals and FMF afflicted men, utilizing previously collected data from PhyloChip™ DNA microarray experiments. A total of 15 healthy and 15 FMF male volunteers, aged 18 to 50, participated in this partially randomized placebo trial, which is accessible through the GEO Series accession number GSE111835.

**Results and Discussion:**

Key findings from current investigations include *i.* the anticipated divergence in gut bacteria resistance to placebo between healthy and FMF individuals, *ii.* the minor impact of placebo on gut bacterial diversities in healthy individuals, with *Enterobacteriaceae* diversities identified as placebo-resistant among “healthy” gut bacteria, and *iii.* the comprehensive influence of placebo on all bacterial phyla in the gut microbiome of FMF patients, extending to nearly all bacterial genera, except for the resilience of gut *Akkermansia muciniphila* spp. to placebo in FMF patients. This study underscores the susceptibility of *Faecalibacterium*, *Blautia*, and *Clostridium* genera to placebo. Consequently, this investigation holds significance for the proper design of placebo-controlled trials and establishes a foundation for further exploration of the gut-brain axis. Furthermore, it contributes valuable insights to discussions regarding proposals for probiotic therapies, particularly focusing on *Faecalibacterium* spp., *Blautia* spp., and *Clostridium* spp.

## Introduction

Clinical trials involving healthy controls face challenges in both subject recruitment and result interpretation (Johnson et al., 2016). Additionally, the orchestration of placebo-controlled trials is a complex process (Howick, 2017), particularly in developing countries (Lepage et al., 2023; Kim et al., 2023). The success of clinical trials is influenced by diverse factors (Kupersmith and Jette, 2023; Jacobsen et al., 2023; Feldman et al., 2022), and despite the abundance of such trials, there remains a need to elucidate placebo effects in both healthy individuals and patients, particularly in studies related to the gut microbiota (Kleine-Borgmann et al., 2023).

Familial Mediterranean Fever (FMF) is a monogenic autosomal recessive autoinflammatory disorder resulting from mutations in the MEFV gene. The disease is characterized by inflammatory episodes affecting serous membranes, leading to periodic fevers and pain (Pepoyan et al., 2015; Pepoyan et al., 2015; Talerico et al., 2020; Bhatt and Cascella, 2023; Gallego et al., 2023; Zadeh et al., 2011). In the context of FMF, placebos are utilized to assess the effects of various drugs and functional foods, including probiotics [beneficial bacteria for humans (Pepoyan and Trchounian, 2009; Pepoyan et al., 2018b)/animals/plants (Manvelyan et al., 2023; Pepoyan et al., 2019a)]. While it is hypothesized that diets rich in antioxidants and supplements with anti-inflammatory properties may partially alleviate FMF symptoms and enhance the well-being of patients, research findings in this realm are contentious (Mansueto et al., 2022; Damián et al., 2022; Mazzantini et al., 2021). The number of clinical trials on FMF is notably high, given the monogenic nature of the disease (Welzel et al., 2021; Tsaturyan et al., 2023; Pepoyan et al., 2018a; Ataya et al., 2023).

The composition of microbiomes is influenced by various factors, including host genetics (Lighthouse et al., 2004; Qin et al., 2022; Boccuto et al., 2023; Pepoyan et al., 2020a), prevailing diseases (Lighthouse et al., 2004; Qin et al., 2022; Boccuto et al., 2023; Pepoyan et al., 2020a), environmental conditions (Berg et al., 2020; Zengler et al., 2019; Kozhakhmetov et al., 2023), and the inherent self-assembly properties of microbes (van Leeuwen et al., 2023; Hovnanyan et al., 2015).

Numerous pieces of evidence suggest a nuanced interplay between microbiota perturbations and the phenotypic expressions of FMF, with the complexity of this relationship influenced by both genetic and environmental factors. The modulation of gut microbiota, encompassing the investigation of probiotic treatments, holds promise for advancing our understanding and management of FMF (Di Ciaula et al., 2020).

Nevertheless, there is ongoing controversy regarding the ability of probiotic treatment to alter the composition of the host microbiota. The health benefits associated with probiotics may arise from the metabolites produced by the bacteria and their interactions with the host’s immune system (Singh et al., 2023). Notably, probiotics have shown the ability to influence gene expression, exerting potential anti-inflammatory effects within the gut microbiota without inducing changes in composition (Ng et al., 2023).

In Armenia, a significant number of male patients with Familial Mediterranean Fever (FMF) has been reported (Tsaturyan et al., 2023). Additionally, there are documented impairments in the host-gut microbiota relationship in FMF disease (Tsaturyan et al., 2023). Moreover, the impact of the probiotic *Lactobacillus acidophilus* strain INMIA 9602 Er 317/402 on the gut microbiota composition of male FMF patients was demonstrated through a placebo/probiotic comparative analysis (Pepoyan et al., 2018a). However, existing data and analyses concerning the effects of placebo on the gut microbiota are limited and do not provide a comprehensive understanding of the placebo’s influence on the overall bacterial composition of the gut microbiota.

The primary objective of this study is to assess the effects of a placebo on the composition of the gut microbiota in male FMF patients. The central research question aims to determine whether there exists a significant diversity of bacteria independent of the placebo effect that can be directly utilized in clinical and nutritional trials.

## Materials and methods

In this study, for the first time, the effect of placebo on the gut microbiota of healthy and FMF men was fully analyzed by leveraging the prior PhyloChip™ DNA-microarray-based data (Tsaturyan et al., 2023; Pepoyan et al., 2018a). Healthy and FMF male volunteers (15/15) aged 18 to 50 took part in this partially randomized placebo trial accessible through GEO Series accession number GSE111835 (Tsaturyan et al., 2023; Pepoyan et al., 2018a) in which the participant took an empty capsule twice daily as a placebo for 1 month. The patients also took their main drug, 1 mg colchicine, as usual. All patients’ diagnoses were confirmed by genetic analysis. None of the study participants had been treated with antibiotics, probiotics, hormones, or chemotherapeutic agents during the month leading up to the study. The duration of the colchicine treatment by patients was more than 7 years. Patients in the acute phase were not included in the study.

Standardization protocols of DNA isolation were implemented to enhance the reliability and comparability of gut microbiome analyses. Samples’ metadata, such as date, time, and participant information, were properly documented and stored. Stool samples collected by volunteer subjects in sterile plastic bags and transported to the laboratory were studied. In order to obtain optimal yield and quality of DNA, both ZR Fecal DNA MiniPrep™ (Zymo Research Corp., Irvine, CA, USA) and Ultraclean^®^ Fecal DNA Isolation (MoBio Laboratories Inc., Carlsbad, CA, USA) commercially available kits were used to isolate total DNA. Chosen DNA extraction kits have been validated for fecal samples. To ensure that the entire sample is homogenized consistently to obtain representative microbial DNA, the bead beating method (FastPrep-24, MP Biomedicals, USA) was used. The extracted DNA was quantified using an absorbance-based method (NanoDrop Microvolume Spectrophotometers, Thermo Fisher, USA). DNA quality was assessed using gel electrophoresis. gDNA was extracted/amplified (16S rRNA gene) from fecal materials frozen at -80^о^C. The primer sequences used for microarrays and 16S rRNA clone libraries were: 27f.jgi (Bacteria-specific) 5′-AGAGTTTGATCCTGGCTCAG-3′ and 1492r.jgi (Bacteria/Archaea-specific) 5′-GGTTACCTTGTTACGACTT-3′.

Bacterial communities were identified using a third-generation, culture-independent, high-density DNA microarray analysis (PhyloChip™; Affymetrix, Santa Clara, CA, USA), according to the investigations described previously (Tsaturyan et al., 2023; Pepoyan et al., 2018a).

This method also enables the estimation of differences in the relative abundance of bacterial taxa based on differences in their hybridization intensities (Kellogg et al., 2013).

In this study, pharmaceutical-grade empty hard-gelatin capsules sourced from Vitamax E, LLC in Yerevan, Armenia, were employed. These gelatin capsules, recognized for their swift absorption in the gastrointestinal tract and their lack of side effects, adhere to GMP, USP, and SP standards. The study participants were unaware that the placebo capsules were empty.

Student’s t-test and Mann-Whitney test were used for statistical analyses. *P <* 0.05 was considered statistically significant. Multibase 2015 Excel Add-in program (NumericalDynamics, Tokyo, Japan) was also used in the prior studies (Tsaturyan et al., 2023; Pepoyan et al., 2018a).

## Results

### Comparative analysis of gut microbiota composition of non-FMF and FMF men before and after the placebo administration: bacterial diversities

A comparative analysis of gut microbiota composition was conducted for non-FMF and FMF men before and after placebo administration, focusing on bacterial diversities. The evaluation covered 18,725 bacterial Operational Taxonomic Units (OTUs) to identify variations in gut bacterial diversities.

In the non-FMF male group, the analysis revealed that 140 OTUs exhibited statistically significant differences after the administration of the placebo (*P <* 0.05). The altered bacteria primarily belonged to the phyla *Firmicutes* (78 OTUs), *Bacteroidetes* (17 OTUs), *Proteobacteria* (16 OTUs), and *Tenericutes* (9 OTUs) (**Table 1**).

**Table 1:** Number of different OTUs after taking the placebo: bacterial phyla*.

Bacterial phyla	Healthy (non-FMF men) OTUs	FMF men OTUs
*Firmicutes*	78 (55.71)	4,777 (63.18)
*Bacteroidetes*	17 (12.14)	350 (4.62)
*Proteobacteria*	16 (11.43)	1,360 (17.99)
*Tenericutes*	9 (6.43)	119 (1.57)
*Actinobacteria*	5 (3.57)	23 (0.304)
Other	15 (10.72)	929 (12.34)
Sum	140	7,560

*The impact of the placebo on 18,725 bacterial OTUs was evaluated; P < 0.05.

In parentheses- OTUs percentages.

FMF, familial Mediterranean fever.

OTUs, operational taxonomic units.

Similarly, in the FMF male group after placebo administration, 7,560 OTUs were altered in the gut microbiota of patients. Among these, 4,777 belonged to *Firmicutes*, 1,360 to *Proteobacteria*, 350 to *Bacteroidetes*, 119 to *Tenericutes*, 23 to *Actinobacteria*, and the remainder to other bacterial phyla (*P <* 0.05) (**Table 1**).

### Impact of placebo: differences in bacterial diversities of *Firmicutes*

The impact of placebo on bacterial diversities within the *Firmicutes* phylum revealed distinctive patterns in non-FMF and FMF men.

For non-FMF men, *Firmicutes* OTUs constituted 55.71% of all different bacterial OTUs, with prominent differences in the order *Clostridiales* (66 OTUs). These differences primarily comprised OTUs from families *Clostridiaceae* (6 OTUs), *Lachnospiraceae* (16 OTUs), and *Ruminococcaceae* (40 OTUs, mostly from an unclassified genus, and 7 OTUs from the genus *Faecalibacterium*). Additionally, 7 OTUs were from the order *Lactobacillales*, and 5 OTUs were from *Bacillales* (**Tables 2**, **3**).

**Table 2:** Number of different OTUs after taking the placebo: Firmicutes*.

Phylum Firmicutes: orders	Healthy (non-FMF men) OTUs	FMF men OTUs
*Clostridiales*	66 (85)	3,969 (83)
*Lactobacillales*	7 (9)	500 (10.99)
*Bacillales*	5 (6)	265 (5.99)
Sum	78	4,777

*The impact of the placebo on 18,725 bacterial OTUs was evaluated; P < 0.05.

In parentheses- OTUs percentages.

FMF, familial Mediterranean fever.

OTUs, operational taxonomic units.

**Table 3:** Number of different OTUs after taking the placebo: Clostridiales*.

Order Clostridiales: families	Healthy (non-FMF men) OTU	FMF men OTU
*Ruminococcaceae*	40 (63.61) (mostly from the unclassified genus, and 7 OTUs from the genus *Faecalibacterium*)	1,365 (35.39)
*Lachnospiraceae*	16 (26.51)	2,086 (54.96)
*Clostridiaceae*	6 (9.88)	361 (9.65)
Sum	66	3,969

*The impact of the placebo on 18,725 bacterial OTUs was evaluated; P < 0.05.

In parentheses- OTUs percentages.

FMF, familial Mediterranean fever.

OTUs, operational taxonomic units.

For FMF men, *Firmicutes* OTUs constituted 63.18% of all different bacterial OTUs, with predominant differences in the order *Clostridiales* (83%) (**Table 2**).

Analysis from **Table 3** indicates that the families *Ruminococcaceae* and *Lachnospiraceae* were particularly susceptible to placebo within the *Firmicutes* phylum. Following placebo administration, the numbers of altered OTUs from the families *Ruminococcaceae* for non-FMF men and FMF men were 40 (63.61%) and 1365 (35.39%), respectively. Similarly, the numbers of altered OTUs from the families *Lachnospiraceae* for non-FMF men and FMF men were 16 (26.51%) and 2086 (54.96%), respectively (**Table 3**).

### Impact of placebo: differences in bacterial diversities of *Bacteroidetes*

The impact of the placebo on the differences in bacterial diversities within the phylum *Bacteroidetes* was examined, highlighting distinctions between non-FMF and FMF men.

In non-FMF men, among *Bacteroidetes* OTUs, which comprised 12.14% of all different bacterial OTUs (**Table 1**), the prominent differences were associated with OTUs of the *Bacteroidia* class, making up 70.59% of the phylum (**Table 4**).

**Table 4:** Number of different OTUs after taking the placebo: *Bacteroidetes* *.

Bacteroidetes: classes	Healthy (non-FMF people)	FMF people
*Bacteroidia*	12 (70.59) (prevailed family: *RikenellaceaeII*- 8 OTUs)	277 (79.44) (prevailed families: *Prevotellaceae*: 84 OTUs *Bacteroidaceae*- 80 OTUs *RikenellaceaeII*- 64 OTUs *Porphyromonadaceae*- 24 OTUs)
*Flavobacteria*	1 (5.88)	37 (11.57)
*Sphingobacteria*	4 (23.53)	32 (8.99)
Sum	17	350

*The impact of the placebo on 18,725 bacterial OTUs was evaluated; P < 0.05.

In parentheses- OTUs percentages.

FMF, familial Mediterranean fever.

OTUs, operational taxonomic units.

For FMF men, among *Bacteroidetes* OTUs, accounting for 4.62% of all different bacterial OTUs (**Table 1**), the significant differences were linked to OTUs of the *Bacteroidia* class, constituting 79.44% of the phylum (**Table 4**). Following placebo administration, OTUs from the families of *Prevotellaceae* (30.32%), *Bacteroidaceae* (28.88%), and *Rikenellaceae*II (23.10%) emerged as the quantitatively dominant different bacterial families within the class (**Table 4**).

### Impact of placebo: differences in bacterial diversities of *Proteobacteria*

In non-FMF men, among *Proteobacteria* OTUs, constituting 11.43% of all different bacterial OTUs (**Table 1**), the predominant differences were associated with OTUs from the following classes: 43.75% *Betaproteobacteria*, 25% *Alphaproteobacteria*, and 25% *Gammaproteobacteria*. It is noteworthy that all different *Gammaproteobacteria* OTUs belonged to the genus *Pseudomonas* (*P <* 0.05) (**Table 5**). Interestingly, there were no placebo-induced alterations in bacterial diversities within *Enterobacteriaceae*.

**Table 5:** Number of different OTUs after taking the placebo: Proteobacteria*.

Proteobacteria*: families*	Healthy (non-FMF people) OTUs	FMF people OTUs
*Enterobacteriaceae*	0	536 (39.41)
*Aquabacteriaceae*	2 (12.5)	175 (12.87)
*Comamonadaceae*	2 (12.5)	81 (5.6)
*Pseudomonadaceae*	4 (25.0)	76 (5.59)
*other*	8 (50)	492 (35.83)
Sum	16	1,360

*The impact of the placebo on 18,725 bacterial OTUs was evaluated; P < 0.05, and the diversities with comparatively large numbers were considered.

In parentheses- OTUs percentages.

FMF, familial Mediterranean fever.

OTUs, operational taxonomic units.

For FMF men, among *Proteobacteria* OTUs, accounting for 17.99% of all different bacterial OTUs (**Table 1**), the predominant differences were related to OTUs of the *Enterobacteriaceae*, constituting 39.41% of the different *Proteobacteria* (*P <* 0.05) (**Table 5**).

### Impact of placebo on hybridization scores of different bacterial diversities

In line with the substantial number of distinct OTUs recorded in FMF disease (**Table 1**), the hybridization scores of bacterial OTUs in FMF individuals after placebo were significantly greater than those of non-FMF individuals:

— *Ruminococcus* spp.: 16 100,866 ± 3 309,224 vs. 531,574 ± 8,309.14
— *Lachnospiraceae* spp.: 17 861,159 ± 4 625,717.4 vs. 200,364.3 ± 3,952.3
— representatives from the *Bacteroidia*: 1 874,301 ± 554,143.2 vs. 146,473.8 ± 8,583.8.

**Figure 1:**
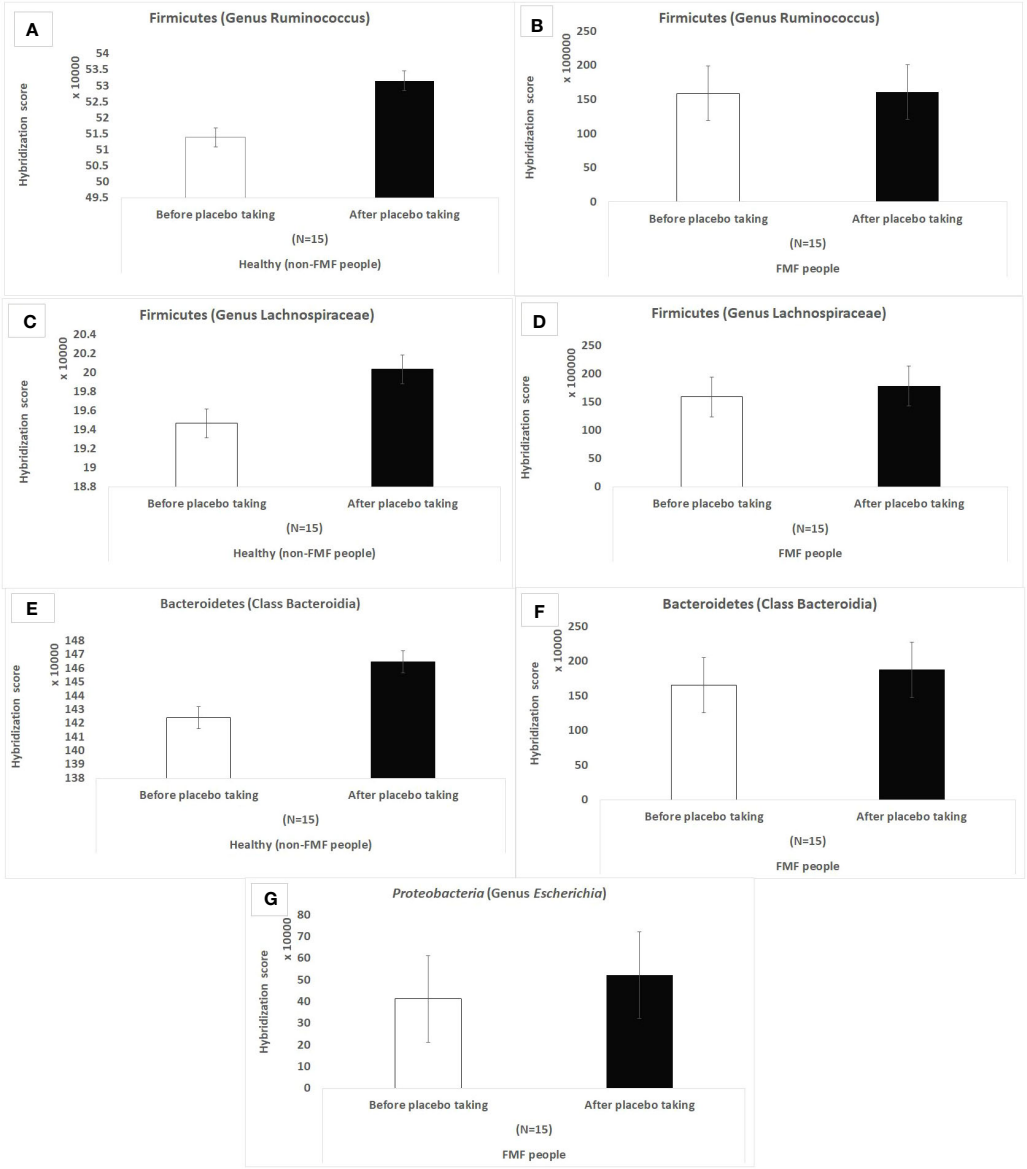
Hybridization scores of altered gut bacterial diversities in non-FMF and FMF men after the placebo administration. The impact of the placebo on 18,725 bacterial OTUs was evaluated; *P* < 0.0001. FMF – Familial Mediterranean fever. OTUs – operational taxonomic units. **(A)** Hybridization score of *Ruminococcus* OTUs after placebo in non-FMF men. **(B)** Hybridization score of *Ruminococcus* OTUs after placebo in FMF men. **(C)** Hybridization score of *Lachnospiraceae* OTUs after placebo in non-FMF men. **(D)** Hybridization score of *Lachnospiraceae* OTUs after placebo in FMF men. **(E)** Hybridization score of *Bacteroidia* OTUs after placebo in non-FMF men. **(F)** Hybridization score of *Bacteroidia* OTUs after placebo in FMF men. **(G)** Hybridization score of *Escherichia* OTUs after placebo in FMF men.

According to the results of the hybridization scores, in the non-FMF male subjects, the placebo produced quantitative changes in the altered main bacterial diversities, which were not observed in the FMF male subjects. There was an increase in *Ruminococcus* spp (513,870.3 ± 10,018.57 vs. 531,574 ± 8,309.14; *P <* 0.05) and *Lachnospiraceae* spp. (194,678 ± 4,802.6 vs. 200,364.3 ± 3,952.3, *P <* 0.05), as well as representatives of *Bacteroidia* after the placebo intake in non-FMF men (**Figure 1**).

### Overlapping gut bacterial diversities in non-FMF and FMF men after the placebo administration (number of OTUs)

**Figure 2:**
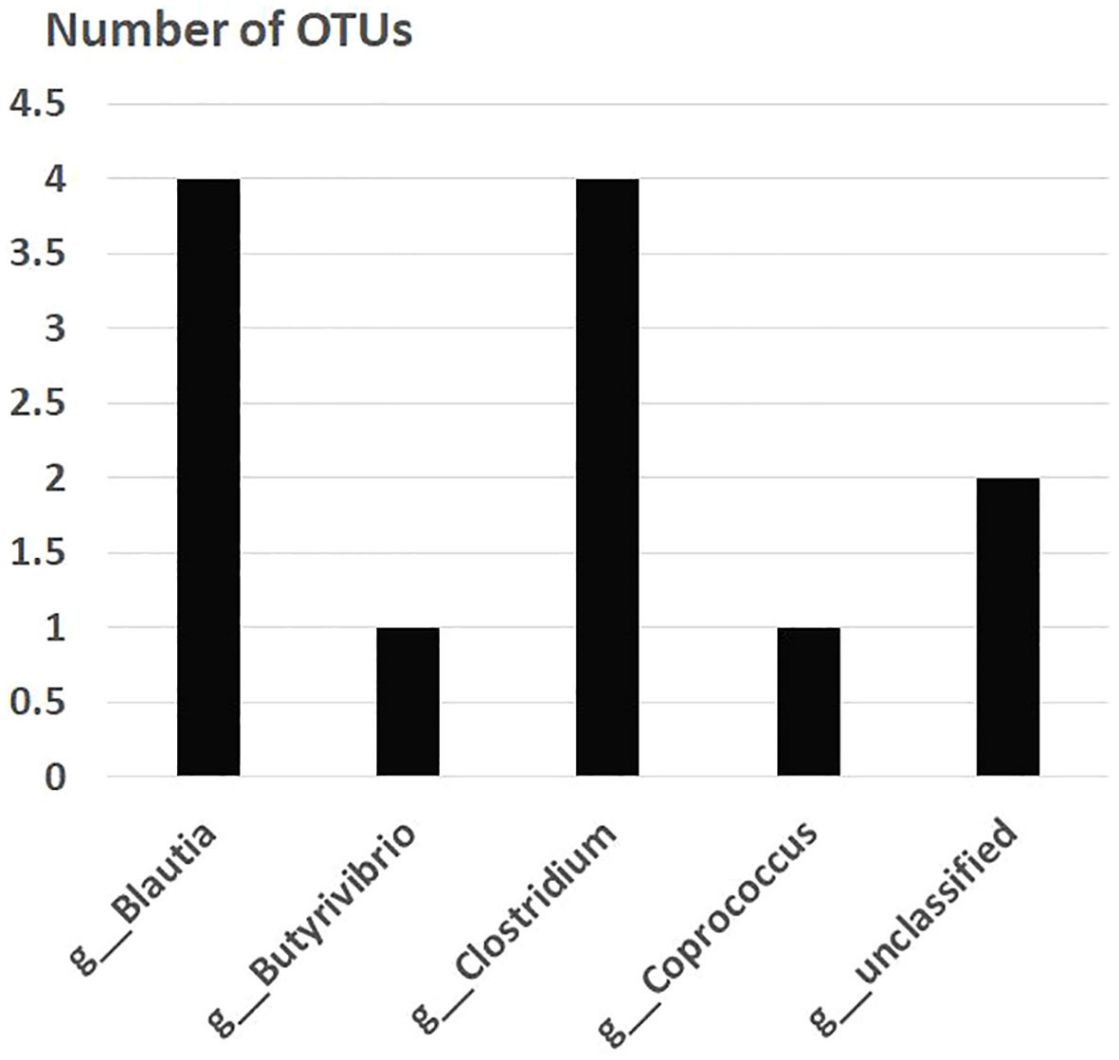
Overlapping gut bacterial diversities of family *Lachnospiraceae* in non-FMF and FMF men after the placebo administration (number of OTUs). *P* < 0001. FMF, Familial Mediterranean fever; OTUs, operational taxonomic units; g, genus.

**Figure 3:**
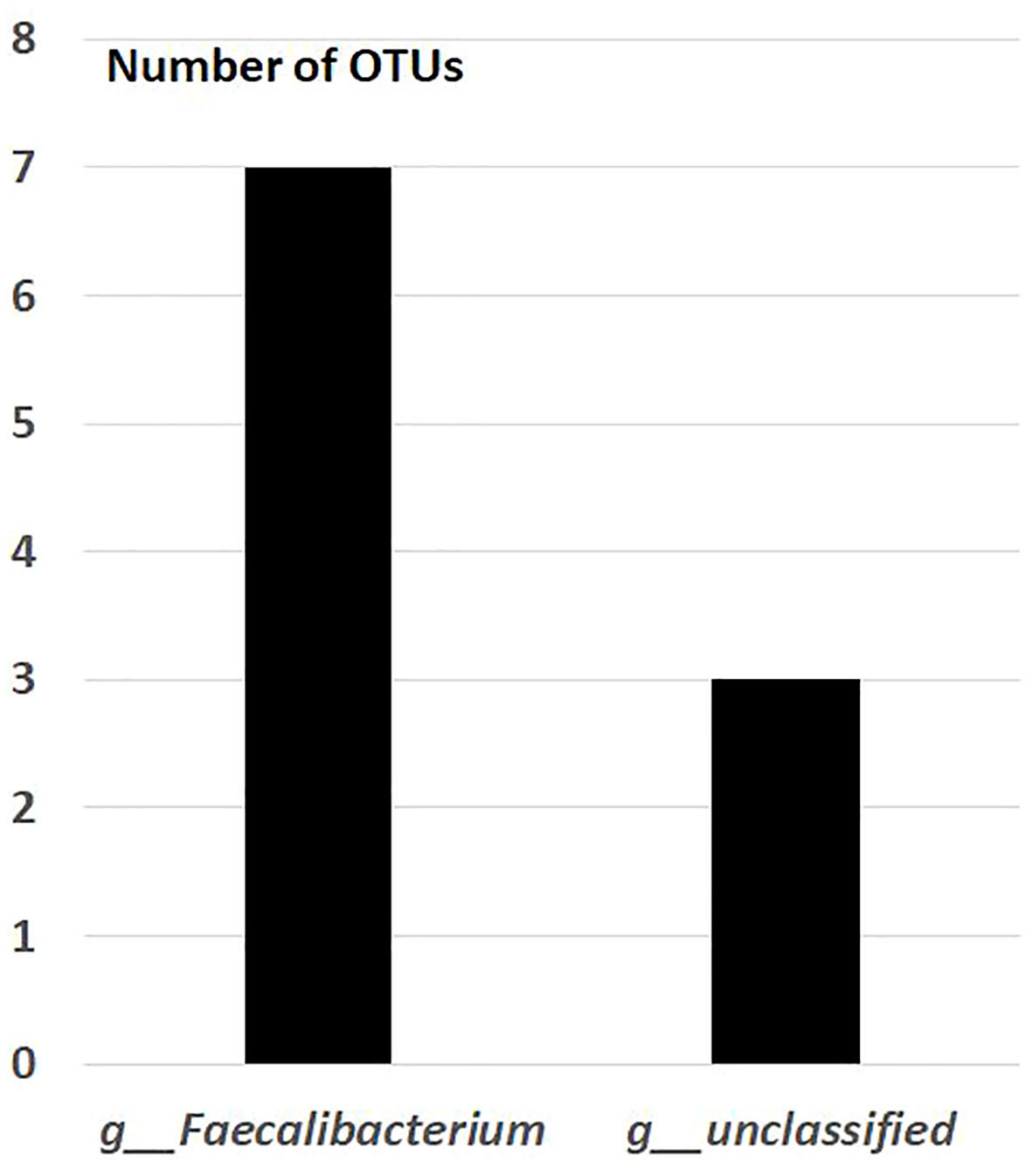
Overlapping gut bacterial diversities of family *Ruminococcaceae* in non-FMF and FMF men after the placebo administration (number of OTUs). *P* < 0001. FMF, Familial Mediterranean fever; OTUs, operational taxonomic units; g, genus; f, family.

After the placebo administration, a total of 54 overlapping gut bacterial diversities were identified from the pool of 18,725 bacterial OTUs in both non-FMF and FMF men. These 54 OTUs were primarily affiliated with the following families:

— *Lachnospiraceae* (12 OTUs) (**Figure 2**).
— *Ruminococcaceae* (10 OTUs) (**Figure 3**).

### Differences in order *Clostridiales* diversities in non-FMF men after the placebo administration

Differences in order *Clostridiales* diversities were noted in non-FMF men after the placebo administration. According to **Table 3**, the number of distinct OTUs for the families *Ruminococcaceae, Lachnospiraceae*, and *Clostridiaceae* spp. after the placebo intake was 40, 16, and 6, respectively, for non-FMF men. When comparing this data with the information on “overlapping gut bacterial diversities in non-FMF and FMF men after the placebo administration,” it suggests that there were specific diversities of bacteria within the *Clostridiales* order that changed after the placebo course in healthy individuals but not in patients.

### Resistance to placebo gut bacterial diversities in non-FMF and FMF men (number of OTUs)

The investigations found that FMF patients did not have unaffected bacterial genera after the placebo course. A limited number of changes were observed in the OTUs related to the genera of *Akkermansia*. Out of the 217 OTUs belonging to the genus *Akkermansia* (phylum: *Verrucomicrobia*), only 22 changed after the placebo administration (**Table 6**). Notably, at the species level, no changes were observed in OTUs related to *Akkermansia muciniphila* with all 108 OTUs remaining unaffected after the placebo course.

**Table 6:** Number of OTUs of resistant to placebo bacterial diversities.

Bacterial diversities	FMF people, N=15		Non-FMF people, N=15	
	Before taking placebo	After taking placebo	Before taking placebo	After taking placebo
*Akkermansia* (phylum *Verrucomicrobia*)	217	195 (89.86) (*P <* 0.05)	237	235 (99.12) (*P <* 0.05)
*A. muciniphila*	108	108 (100) (*P <* 0.05)	107	105 (95.33) (*P <* 0.05)
*Enterobacteriaceae*	1,228	686 (55.86)	0	0

*The impact of the placebo on 18,725 bacterial OTUs was evaluated; P < 0.05.

In parentheses- OTUs percentages.

FMF, familial Mediterranean fever.

OTUs, operational taxonomic units.

P, comparison of data (before and after placebo administration for the group).

## Discussion

Approximately 1,500 bacterial species, spanning over 50 different phyla within the intestinal microbiota, play a crucial role in maintaining normal human physiology and health (Conz et al., 2023). The colonic microbiota, boasting the greatest diversity, harbors up to 100 trillion bacteria. In the symbiotic relationship between bacteria and the host, gut bacteria collaborate with the host to ensure the well-being of the nervous system as well (Morais et al., 2021). The human gut microbiome exhibits gender-specific characteristics (Pepoyan et al., 2021; Bardhan and Yang, 2023) and potential interindividual variations (Chen et al., 2022; Afzaal et al., 2022; Wan et al., 2023). The link between the intestinal and systemic immune systems is primarily influenced by the expansion of the immune response through lymphatic and blood circulation (Zheng et al., 2020; Campbell et al., 2023; Li et al., 2022).

Currently, a wealth of data supports the notion that disruptions in the diversity of human gut bacteria, particularly a low level of bacterial diversity within the genus *Faecalibacterium*, can lead to undesirable consequences, such as inflammatory processes (Martín et al., 2023). In instances of inflammatory or metabolic diseases, a reduction in bacterial diversities is also noted in *Blautia* (Liu et al., 2021) and *Clostridium* (Guo et al., 2020). This may explain the extensive discussions surrounding the potential use of these bacteria as next-generation probiotics or living biotherapeutics (Martín et al., 2023; Guo et al., 2020).

Conversely, in the context of metabolic diseases, there is a growing focus on mucin-degrading species of *A. muciniphila* from the *Verrucomicrobia* phylum. This species has recently garnered significant attention and is widely discussed as a potential candidate for next-generation probiotics (Jian et al., 2023).

Preliminary evidence suggests the potential clinical utility of probiotics for FMF. Specifically, studies have demonstrated that a formulation containing eight bacterial strains, known as the De Simone Formulation and marketed as Vivomixx^®^ in Europe and Visbiome^®^ in the US, may have beneficial effects when administered during the inter-critical period of FMF. This intervention shows promise in improving symptoms, particularly in a subgroup of FMF patients characterized by more severe disease and partial resistance to colchicine (Di Ciaula et al., 2020).

Additionally, our prior investigations evidence the impact of probiotic Narine (*Lactobacillus acidophilus* INMIA 9602 Er-2 strain 317/402) on the disease manifestation. Specifically, it has been shown that intake of *Lactobacillus acidophilus* INMIA 9602 Er-2 strain 317/402 has positive effects, including the normalization of serum C-reactive protein levels in FMF patients during remission (Pepoyan et al., 2015; Pepoyan et al., 2017; Balayan et al., 2015).

In addition to analyzing blood parameters, our previous studies also delved into the impact of the probiotic Narine on the composition of specific members of the intestinal microbiota in patients with FMF (Pepoyan et al., 2018a).

In the context of FMF disease (Pepoyan et al., 2017; Balayan et al., 2015; Manzano et al., 2023; Touitou and Pepoyan, 2008; Lancieri et al., 2023; Pepoyan et al., 2019b), placebos are employed to evaluate the impacts of probiotics [microorganisms that confer beneficial effects on humans (Pepoyan et al., 2023; García-Santos et al., 2023; Harutyunyan et al., 2022), animals (Ataya et al., 2023; Balayan et al., 2019; Rodriguez et al., 2017; Wang et al., 2023; Mirzabekyan et al., 2023; Šefcová et al., 2023), and plant host metabolism (Rahman et al., 2018; Pepoyan and Chikindas, 2020; Mockevičiūtė et al., 2023)], along with their metabolites, as well as medications in general (Ben-Zvi et al., 2017; Haviv and Hashkes, 2016; Hashkes and Huang, 2015; Hashkes et al., 2014). While placebos have long served as inert controls in clinical trials (Gupta and Verma, 2013; Finniss et al., 2010; Louhiala and Puustinen, 2017), it is essential to recognize that placebo effects are psychobiological phenomena (Pogany, 2017; Hashmi, 2018; Liu, 2022; Schaefer et al., 2023; Shafir et al., 2023) capable of producing effects similar to certain drugs, even when patients are not knowingly given placebos (Bräscher et al., 2022). The term “placebo” originates from the Latin word “placere” (Schaefer et al., 2023; Yetman et al., 2021), meaning “to please” (Dreber et al., 2023). Surprisingly, approximately 40% of medications exhibit placebo effects (Sonawalla and Rosenbaum, 2002; Fässler et al., 2010; Pardo-Cabello et al., 2022; Moerbeek, 2023).

A “pure” placebo is typically represented by empty capsules (Welzel et al., 2021; Tsaturyan et al., 2023; Franc et al., 2022; Moerbeek, 2023) or inert substances like starch, dextromaltose, lactose, talc, mentholated water, and saline (Mitsikostas et al., 2020). The diversity of placebo effects is attributed to various biological mechanisms, influenced by the evolutionary development of the body’s unique defense mechanisms (Benedetti, 2014; Buergler et al., 2023; Seneviratne et al., 2022; Pronovost-Morgan et al., 2023). Despite the literature data on placebo-dependent studies on the gut microbiota of FMF patients, in these studies, the placebo effect is discussed in part, depending on the nature of the problems presented in the articles. Perhaps, it was these incomplete discussions that pointed to the need for a full discussion of placebo effects on the gut microbiota of FMF patients. This study revealed distinct effects of a placebo on the bacterial diversities of the gut microbiota in both healthy and FMF-afflicted men, with a more pronounced impact observed in those with FMF.

Analysis of hybridization scores indicated that in non-FMF male subjects, the placebo induced quantitative changes in the altered main bacterial diversities, a phenomenon not observed in FMF male subjects.

### Overlapping gut bacterial diversities in non-FMF and FMF men after the placebo administration

Considering the changed bacterial diversities observed in both healthy and diseased volunteers across studies, the overlapping gut bacterial variations should be carefully considered in placebo-dependent FMF-gut microbiota studies. Dysbiosis and inflammation in the gut have been associated with various mental illnesses, including prevalent conditions like anxiety and depression (Clapp et al., 2017). Moreover, the impact of gut bacteria on anxiety and depression levels appears to be influenced by gender (Pepoyan et al., 2021). It is conceivable that the overlapping gut bacterial diversities observed in non-FMF and FMF men after placebo administration represent key bacterial varieties with potential beneficial effects on anxiety and depression levels in both groups.

During the investigation, interviews were conducted to assess the anxiety and depression levels of the participants (Pepoyan et al., 2021). Even simple, non-test interviews indicated that after the placebo administration, both healthy individuals using the “pills” as an immunostimulant and patients felt more resilient to various infections and perceived themselves as healthier than before taking the “pills.” This observation was supported by the placebo’s effect on the psychoemotional status of men, potentially influenced by corresponding changes in intestinal bacteria.

The present research underscores the sensitivity of several species within the *Faecalibacterium, Blautia*, and *Clostridium* genera to the placebo effect. The significance of F*aecalibacterium* (Martín et al., 2023), *Blautia* (Liu et al., 2021), and *Clostridium* spp (Guo et al., 2020). in inflammatory/metabolic diseases is well-established. The observation that *Faecalibacterium*, *Blautia*, and *Clostridium* are influenced by the placebo effect could have noteworthy implications for clinical studies, particularly within the field of microbiome research. Clinical trials involving interventions that impact these bacteria must carefully consider the placebo effect, especially when evaluating the effectiveness of treatments targeting specific microbiota for diseases. It is crucial to understand how the placebo, including the type of capsule used (e.g., empty gelatin capsule), may affect these bacteria to accurately assess treatment outcomes. These findings are also significant for discussions regarding the potential use of *Faecalibacterium*, *Blautia*, and *Clostridium* spp. as probiotics.

These positive changes due to the placebo effect likely indicate that despite claims that it is ethically wrong to deceive people with placebos, it is still possible to prescribe placebos in extreme circumstances (for example, drug shortages).

### Differences in gut bacterial diversities in non-FMF men after the placebo administration

Care should be exercised in interpreting the changes in bacterial diversities that occurred after the placebo course, especially when comparing healthy individuals and patients. In the presented study, it has been shown that there were bacterial diversities that changed after the placebo course in healthy individuals but not in patients.

This observation warrants further investigation. It is possible that the dietary habits of individuals with FMF may also influence the placebo effect (Mansueto et al., 2022).

### Differences in gut bacterial diversities in FMF men after the placebo administration

As mentioned, the association between genetics and gut microbiota was recognized in FMF patients (Tsaturyan et al., 2023; Pepoyan et al., 2018a). Following a placebo course in FMF patients, substantial changes were observed compared to healthy individuals. While some of these changes may be influenced by factors present in both healthy individuals and FMF patients, it was evident that these alterations should be duly considered in the design and interpretation of future clinical trials focused on gut microbiota in FMF patients.

### Bacterial diversities that did not undergo changes after placebo

The investigations have uncovered that no bacterial genera were left unaffected after the placebo course in FMF patients. Conversely, there were no placebo-induced altered bacterial differences observed in *Enterobacteriaceae* diversities for non-FMF men. The *Enterobacteriaceae* spp. encompasses both pathogenic and commensal bacterial diversities, including commensal *Escherichia coli* (Tsaturyan et al., 2022; Pepoyan et al., 2020b). Prior research has highlighted that the prevalence of dominant commensal *E. coli* in the gut can vary depending on the health status of an individual (Shahinyan et al., 2003; Stepanyan et al., 2007; Mirzoyan et al., 2006; Pepoyan et al., 2014). In a study on *E. coli* isolates in colorectal cancer patients, Tang and colleagues concluded that “diseased” isolates suppressed the growth of healthy isolates under nutrient-limited culture conditions (Shandilya et al., 2021). This effect is possibly linked to altered gut-microbiota-mediated oxidative stress (Pepoyan et al., 2020b; Ni et al., 2022), a phenomenon also observed during FMF disease (Pepoyan et al., 2017).

These studies once again underscore the existence of a gut-brain connection.

## Limitations of the study

Considering the qualitative changes in the microflora found during our research and the limitations that could affect the results of the research, more global studies including a larger number of participants have been planned to be conducted. Although the use of DNA-microarray-based data for analyzing gut microbiota is a powerful tool, it comes with potential biases and limitations (e.g., detection limitations of low abundance species, reference database bias, and limited quantitative accuracy). To address these limitations, using sequencing and quantitative PCR methods in further research to assess more clearly the qualitative and quantitative composition of the microflora is planned.

## Conclusion

This study addresses the escalating demand for placebo-controlled trials by synthesizing knowledge on their impact on human gut microbiota, particularly in FMF patients. Beyond existing data on FMF patients, the focus is on identifying bacterial diversity unaffected by placebos for reliable use in clinical trials.

Noteworthy findings reveal that gut bacteria in healthy and FMF patients differ in their response to placebos. In healthy individuals, placebo minimally influences bacterial diversities, altering only 140 of 18,725 examined bacterial OTUs. Despite this limited change, all bacterial phyla are affected, excluding *Enterobacteriaceae* spp., which may be of value for studies involving healthy subjects.

Conversely, placebos affect all gut bacteria phyla in FMF patients, extending to nearly all bacterial genera. *Akkermansia* from the phylum *Verrucomicrobia* shows relative resistance, with only 22 out of 217 OTUs affected. *Faecalibacterium, Blautia*, and *Clostridium* genera exhibit susceptibility to placebo in both FMF and non-FMF men, showcasing distinct diversities altered after placebo administration.

Importantly, the study reveals that placebo-induced quantitative changes in bacterial diversities in non-FMF men differ from FMF male subjects, as indicated by hybridization scores. This study, critical for placebo-controlled trial design, not only lays the groundwork for exploring the gut-brain axis but also informs discussions on probiotic therapies involving *Faecalibacterium* spp., *Blautia* spp., and *Clostridium* spp.

## Data availability statement

The datasets presented in this study can be found in online repositories. The names of the repository/repositories and accession number(s) can be found below: GEO Series accession number GSE111835.

## Ethics statement

The study was approved by the Ethics Committee at the Higher Education and Science Committee of Armenia (10-15-21AG; 21/10/2021).

## Author contributions

EP: Formal analysis, Investigation, Writing – review & editing. FM: Writing – review & editing. AM: Investigation, Validation, Writing – review & editing. AG: Writing – review & editing. LS: Writing – review & editing. HG: Writing – review & editing. LG: Formal analysis, Writing – review & editing. MM: Writing – review & editing. MB: Investigation, Writing – review & editing. NH: Investigation, Writing – review & editing. SM: Investigation, Writing – review & editing. VT: Formal analysis, Supervision, Writing – review & editing. TT: Methodology, Resources, Software, Writing – review & editing. AP: Conceptualization, Data curation, Methodology, Project administration, Supervision, Validation, Visualization, Writing – original draft.

## References

[B1] Afzaal M. Saeed F. Shah Y. A. Hussain M. Rabail R. Socol C. T.. (2022). Human gut microbiota in health and disease: Unveiling the relationship. Front. Microbiol. 13. doi: 10.3389/fmicb.2022.999001 PMC9549250 36225386

[B2] Ataya J. Soqia J. Alfawal M. Kara Tahhan N. Albani N. Hani Y. (2023). Awareness and knowledge of familial Mediterranean fever among medical scope students in Syrian universities: A cross-sectional study. SAGE Open Med. 11, 20503121231155996. doi: 10.1177/20503121231155996 36815136 PMC9940211

[B3] Balayan M. Manvelyan A. Marutyan S. Isajanyan M. Tsaturyan V. Pepoyan A.. (2015). Impact of *Lactobacillus acidophilus* INMIA 9602 Er-2 and *Escherichia coli* M-17 on some clinical blood characteristics of Familial Mediterranean Fever disease patients from the Armenian Cohort. Int. J. Probiot. Prebiot. 10, 91–95.

[B4] Balayan M. Pepoyan A. Manvelyan A. Tsaturyan V. Grigoryan B. Abrahamyan A.. (2019). Combined use of eBeam irradiation and the potential probiotic *Lactobacillus rhamnosus* Vahe for control of foodborne pathogen *Klebsiella pneumoniae* . Ann. Microbiol. 69, 1579–1582. doi: 10.1007/s13213-019-01522-2

[B5] Bardhan P. Yang T. (2023). Sexual dimorphic interplays between gut microbiota and antihypertensive drugs. Curr. Hypertens. Rep. 25, 163–172. doi: 10.1007/s11906-023-01244-6 37199902 PMC10193343

[B6] Ben-Zvi I. Kukuy O. Giat E. Pras E. Feld O. Kivity S.. (2017). Anakinra for colchicine-resistant familial Mediterranean fever: A randomized, double-blind, placebo-controlled trial. Arthritis Rheumatol. 69, 854–862. doi: 10.1002/art.39995 27860460

[B7] Benedetti F. (2014). “A modern view of placebo and placebo-related effects,” in Placebo Effects: 2nd Edition: Understanding the mechanisms in health and disease, 2nd edn (Oxford: Oxford Academic). doi: 10.1093/acprof:oso/9780198705086.003.0002

[B8] Berg G. Rybakova D. Fischer D. Cernava T. Vergès M. C. Charles T.. (2020). Microbiome definition re-visited: old concepts and new challenges. Microbiome 8, 103. doi: 10.1186/s40168-020-00875-0 32605663 PMC7329523

[B9] Bhatt H. Cascella M. (2023). Familial Mediterranean fever. In StatPearls [Internet]. (Treasure Island (FL): StatPearls Publishing). Available at: https://www.ncbi.nlm.nih.gov/books/NBK560754/. 32809589

[B10] Boccuto L. Tack J. Ianiro G. Abenavoli L. Scarpellini E. (2023). Human genes involved in the interaction between host and gut microbiome: Regulation and pathogenic mechanisms. Genes (Basel) 14, 857. doi: 10.3390/genes14040857 37107615 PMC10137629

[B11] Bräscher A. K. Ferti I. E. Witthöft M. (2022). Open-label placebo effects on psychological and physical well-being: A conceptual replication study. Clin. Psychol. Eur. 4, e7679. doi: 10.32872/cpe.7679 36762351 PMC9881123

[B12] Buergler S. Sezer D. Bagge N. Kirsch I. Locher C. Carvalho C.. (2023). Imaginary pills and open-label placebos can reduce test anxiety by means of placebo mechanisms. Sci. Rep. 13, 2624. doi: 10.1038/s41598-023-29624-7 36788309 PMC9926426

[B13] Campbell C. Kandalgaonkar M. R. Golonka R. M. Yeoh B. S. Vijay-Kumar M. Saha P. (2023). Crosstalk between gut microbiota and host immunity: Impact on inflammation and immunotherapy. Biomedicines 11, 294. doi: 10.3390/biomedicines11020294 36830830 PMC9953403

[B14] Chen L. Zhernakova D. V. Kurilshikov A. Andreu-Sánchez S. Wang D. Augustijn H. E.. (2022). Influence of the microbiome, diet and genetics on inter-individual variation in the human plasma metabolome. Nat. Med. 28, 2333–2343. doi: 10.1038/s41591-022-02014-8 36216932 PMC9671809

[B15] Clapp M. Aurora N. Herrera L. Bhatia M. Wilen E. Wakefield S. (2017). Gut microbiota’s effect on mental health: The gut-brain axis. Clin. Pract. 7, 987. doi: 10.4081/cp.2017.987 29071061 PMC5641835

[B16] Conz A. Salmona M. Diomede L. (2023). Effect of non-nutritive sweeteners on the gut microbiota. Nutrients 15, 1869. doi: 10.3390/nu15081869 37111090 PMC10144565

[B17] Damián M. R. Cortes-Perez N. G. Quintana E. T. Ortiz-Moreno A. Garfias Noguez C. Cruceño-Casarrubias C. E.. (2022). Functional foods, nutraceuticals and probiotics: A focus on human health. Microorganisms 10, 1065. doi: 10.3390/microorganisms10051065 35630507 PMC9143759

[B18] Di Ciaula A. Stella A. Bonfrate L. Wang D. Q. Portincasa P. (2020). Gut microbiota between environment and genetic background in Familial Mediterranean Fever (FMF). Genes 11, 1041. doi: 10.3390/genes11091041 32899315 PMC7563178

[B19] Dreber A. Johanneson M. Yang Y. (2023). Selective reporting of placebo tests in top economics journals. Available at SSRN: doi: 10.2139/ssrn.4456494

[B20] Fässler M. Meissner K. Schneider A. Linde K. (2010). Frequency and circumstances of placebo use in clinical practice–a systematic review of empirical studies. BMC Med. 8, 15. doi: 10.1186/1741-7015-8-15 20178561 PMC2837612

[B21] Feldman H. A. Feldman J. A. Miller C. C. Walsh G. Tyson J. E. (2022). Informed consent for placebo-controlled trials: Do Ethics and Science Conflict? Ethics Hum. Res. 44, 42–48. doi: 10.1002/eahr.500142 36047276 PMC9841466

[B22] Finniss D. G. Kaptchuk T. J. Miller F. Benedetti F. (2010). Biological, clinical, and ethical advances of placebo effects. Lancet 375, 686–695. doi: 10.1016/S0140-6736(09)61706-2 20171404 PMC2832199

[B23] Franc A. Vetchý D. Fülöpová N. (2022). Commercially available enteric empty hard capsules, production technology and application. Pharmaceuticals (Basel) 15, 1398. doi: 10.3390/ph15111398 36422528 PMC9696354

[B24] Gallego E. Arias-Merino G. Sánchez-Díaz G. Villaverde-Hueso A. Posada de la Paz M. Alonso-Ferreira V. (2023). Familial mediterranean fever in Spain: Time trend and spatial distribution of the hospitalizations. Int. J. Environ. Res. Public Health 20, 4374. doi: 10.3390/ijerph20054374 36901385 PMC10002354

[B25] García-Santos J. A. Nieto-Ruiz A. García-Ricobaraza M. Cerdó T. Campoy C. (2023). Impact of probiotics on the prevention and treatment of gastrointestinal diseases in the pediatric population. Int J Mol Sci. 29, 9427. doi: 10.3390/ijms24119427 PMC10253478 37298377

[B26] Guo P. Zhang K. Ma X. He P. (2020). *Clostridium* species as probiotics: potentials and challenges. J. Anim. Sci. Biotecnol. 11, 24. doi: 10.1186/s40104-019-0402-1 PMC7031906 32099648

[B27] Gupta U. Verma M. (2013). Placebo in clinical trials. Perspect. Clin. Res. 4, 49–52. doi: 10.4103/2229-3485.106383 23533982 PMC3601706

[B28] Harutyunyan N. Kushugulova A. Hovhannisyan N. Pepoyan A. (2022). One health probiotics as biocontrol agents: One health tomato probiotics. Plants (Basel) 11, 1334. doi: 10.3390/plants11101334 35631758 PMC9145216

[B29] Hashkes P. J. Huang B. (2015). The familial Mediterranean fever (FMF) 50 score: does it work in a controlled clinical trial? Re-analysis of the trial of rilonacept for patients with colchicine-resistant or intolerant FMF. Isr. Med. Assoc. J. 17, 137–140. doi: 10.1186/1546-0096-13-S1-P158. 25946762

[B30] Hashkes P. J. Spalding S. J. Hajj-Ali R. Giannini E. H. Johnson A. Barron K. S.. (2014). The effect of rilonacept versus placebo on health-related quality of life in patients with poorly controlled familial Mediterranean fever. BioMed. Res. Int. 2014, 854842. doi: 10.1155/2014/854842 25147819 PMC4131422

[B31] Hashmi J. A. (2018). “Chapter ten - placebo effect: Theory, mechanisms and teleological roots,” in International Review of Neurobiology, 2nd ed, vol. 139. (Academic Press, Luana Colloca), 233–253. doi: 10.1016/bs.irn.2018.07.017 30146049

[B32] Haviv R. Hashkes P. J. (2016). Canakinumab investigated for treating familial Mediterranean fever. Expert Opin. Biol. Ther. 16, 1425–1434. doi: 10.1080/14712598.2016.1233963 27603969

[B33] Hovnanyan K. Marutyan S. Pepoyan A. Navasardyan L. Trchounian A. (2015). Transmission and scanning electron microscopy of contacts between bacterial and yeast cells in biofilms on different surfaces. Open Access Library J. 2, 1–10. doi: 10.4236/oalib.1101492

[B34] Howick J. (2017). The relativity of ‘placebos’: defending a modified version of Grünbaum’s definition. Synthese 194, 1363–1396. doi: 10.1007/s11229-015-1001-0

[B35] Jacobsen S. M. Moore T. Douglas A. Lester D. Johnson A. L. Vassar M. (2023). Discontinuation and nonpublication analysis of chronic pain randomized controlled trials. Pain Rep. 8, e1069. doi: 10.1097/PR9.0000000000001069 37032814 PMC10079346

[B36] Jian H. Liu Y. Wang X. Dong X. Zou X. (2023). *Akkermansia muciniphila* as a next-generation probiotic in modulating human metabolic homeostasis and disease progression: A role mediated by gut-liver-brain axes? Int. J. Mol. Sci. 24, 3900. doi: 10.3390/ijms24043900 36835309 PMC9959343

[B37] Johnson R. A. Rid A. Emanuel E. Wendler D. (2016). Risks of phase I research with healthy participants: A systematic review. Clin. Trials. 13, 149–160. doi: 10.1177/1740774515602868 26350571 PMC4783291

[B38] Kellogg C. A. Piceno Y. M. Tom L. M. DeSantis T. Z. Gray M. A. Zawada D. G.. (2013). Comparing bacterial community composition between healthy and white plague-like disease states in Orbicella annularis using PhyloChip™ G3 microarrays. PloS One 8, e79801. doi: 10.1371/journal.pone.0079801 24278181 PMC3835879

[B39] Kim E. Yang J. Park S. Shin K. (2023). Factors affecting success of new drug clinical trials. Ther. Innov. Regul. Sci. 57, 737–750. doi: 10.1007/s43441-023-00509-1 37166743 PMC10173933

[B40] Kleine-Borgmann J. Dietz T. N. Schmidt K. Bingel U. (2023). No long-term effects after a 3-week open-label placebo treatment for chronic low back pain: a 3-year follow-up of a randomized controlled trial. Pain 164, 645–652. doi: 10.1097/j.pain.0000000000002752 35947884 PMC9916047

[B41] Kozhakhmetov S. Babenko D. Issilbayeva A. Nurgaziyev M. Kozhakhmetova S. Meiramova A.. (2023). Oral microbial signature of rheumatoid arthritis in female patients. J. Clin. Med. 12, 3694. doi: 10.3390/jcm12113694 37297889 PMC10253734

[B42] Kupersmith M. J. Jette N. (2023). Specific recommendations to improve the design and conduct of clinical trials. Trials 24, 263. doi: 10.1186/s13063-023-07276-2 37038147 PMC10084694

[B43] Lancieri M. Bustaffa M. Palmeri S. Prigione I. Penco F. Papa R.. (2023). An update on familial Mediterranean fever. Int. J. Mol. Sci. 24, 9584. doi: 10.3390/ijms24119584 37298536 PMC10253709

[B44] Lepage S. Conway A. Goodson N. Wicks P. Flight L. Devane D. (2023). Online randomised trials with children: A scoping review. PloS One 18, e0280965. doi: 10.1371/journal.pone.0280965 37228143 PMC10212186

[B45] Li X. Zhang S. Guo G. Han J. Yu J. (2022). Gut microbiome in modulating immune checkpoint inhibitors. EBioMedicine 82, 104163. doi: 10.1016/j.ebiom.2022.104163 35841869 PMC9297075

[B46] Lighthouse J. Naito Y. Helmy A. Hotten P. Fuji H. Min C. H.. (2004). Endotoxinemia and benzodiazepine-like substances in compensated cirrhotic patients: a randomized study comparing the effect of rifaximine alone and in association with a symbiotic preparation. Hepatol. Res. 28, 155–160. doi: 10.1016/j.hepres.2003.11.005 15036072

[B47] Liu T. (2022). Placebo effects: A new theory. Clin. psychol. Sci. 10, 27–40. doi: 10.1177/21677026211009799

[B48] Liu X. Mao B. Gu J. Wu J. Cui S. Wang G.. (2021). *Blautia*-a new functional genus with potential probiotic properties? Gut Microbes 13, 1–21. doi: 10.1080/19490976.2021.1875796 PMC7872077 33525961

[B49] Louhiala P. Puustinen R. (2017). “Meaning and use of placebo: philosophical considerations,” in Handbook of the Philosophy of Medicine. Eds. Schramme T. Edwards S. (Springer, Dordrecht), 717–728. doi: 10.1007/978-94-017-8688-1_34

[B50] Mansueto P. Seidita A. Chiavetta M. Genovese D. Giuliano A. Priano W.. (2022). Familial Mediterranean Fever and diet: A narrative review of the scientific literature. Nutrients 14, 3216. doi: 10.3390/nu14153216 35956392 PMC9370508

[B51] Manvelyan A. Balayan M. Miralimova S. Chistyakov V. Pepoyan A. (2023). Biofilm formation and auto-aggregation abilities of novel targeted aqua-probiotics. Funct. Foods Health Dis. 13, 179–190. doi: 10.31989/ffhd.v13i4.1093

[B52] Manzano G. S. Rice D. R. Zurawski J. Jalkh Y. Bakshi R. Mateen F. J. (2023). Familial Mediterranean fever and multiple sclerosis treated with ocrelizumab.Case report. J. Neuroimmunol. 379, 578099. doi: 10.1016/j.jneuroim.2023.578099 37172371

[B53] Martín R. Rios-Covian D. Huillet E. Auger S. Khazaal S. Bermúdez-Humarán L. G.. (2023). *Faecalibacterium*: a bacterial genus with promising human health applications. FEMS Microbiol. Rev. 47, fuad039. doi: 10.1093/femsre/fuad039 37451743 PMC10410495

[B54] Mazzantini D. Calvigioni M. Celandroni F. Lupetti A. Ghelardi E. (2021). Spotlight on the compositional quality of probiotic formulations marketed worldwide. Front. Microbiol. 12. doi: 10.3389/fmicb.2021.693973 PMC8329331 34354690

[B55] Mirzabekyan S. Harutyunyan N. Manvelyan A. Malkhasyan L. Balayan M. Miralimova S.. (2023). Fish probiotics: Cell surface properties of fish intestinal Lactobacilli and *Escherichia coli* . Microorganisms 11, 595. doi: 10.3390/microorganisms11030595 36985169 PMC10052099

[B56] Mirzoyan N. S. Pepoyan A. Z. Trchounian A. H. (2006). Modification of the biophysical characteristics of membranes in commensal Escherichia coli strains from breast cancer patients. FEMS Microbiol. Lett. 254, 81–86. doi: 10.1111/fml.2006.254.issue-1 16451183

[B57] Mitsikostas D. D. Blease C. Carlino E. Colloca L. Geers A. L. Howick J.. (2020). Federation EH. European Headache Federation recommendations for placebo and nocebo terminology. J. Headache Pain. 21, 117. doi: 10.1186/s10194-020-01178-3 32977761 PMC7519524

[B58] Mockevičiūtė R. Jurkonienė S. Šveikauskas V. Zareyan M. Jankovska-Bortkevič E. Jankauskienė J.. (2023). Probiotics, proline and calcium induced protective responses of *Triticum aestivum* under drought stress. Plants (Basel) 12, 1301. doi: 10.3390/plants12061301 36986989 PMC10051984

[B59] Moerbeek M. (2023). Optimal placebo-treatment comparisons in trials with an incomplete within-subject design and heterogeneous costs and variances. PloS One 18, e0283382. doi: 10.1371/journal.pone.0283382 37079588 PMC10118159

[B60] Morais L. H. Schreiber H. L. Mazmanian S. K. (2021). The gut microbiota–brain axis in behaviour and brain disorders. Nat. Rev. Microbiol. 19, 241–255. doi: 10.1038/s41579-020-00460-0 33093662

[B61] Ng Q. X. Lim Y. L. Yaow C. Y. L. Ng W. K. Thumboo J. Liew T. M. (2023). Effect of probiotic supplementation on gut microbiota in patients with Major Depressive Disorders: A systematic review. Nutrients 15, 1351. doi: 10.3390/nu15061351 36986088 PMC10052013

[B62] Ni Q. Zhang P. Li Q. Han Z. (2022). Oxidative stress and gut microbiome in inflammatory skin diseases. Front. Cell Dev. Biol. 10. doi: 10.3389/fcell.2022.849985 PMC8937033 35321240

[B63] Pardo-Cabello A. J. Manzano-Gamero V. Puche-Cañas E. (2022). Placebo: a brief updated review. Naunyn Schmiedebergs Arch. Pharmacol. 395, 1343–1356. doi: 10.1007/s00210-022-02280-w 35943515 PMC9361274

[B64] Pepoyan A. Z. Balayan M. A. Atrutyunyan N. A. Grigoryan A. G. Tsaturyan V. V. Manvelyan A. M.. (2015). Antibiotic resistance of *E. coli* of the intestinal microbiota in patients with familial Mediterranean fever. Klinicheskaia Med. 93, 37–39. 26596057

[B65] Pepoyan A. Z. Balayan M. H. Malkhasyan L. Manvelyan A. Bezhanyan T. Paronikyan R.. (2019a). Effects of probiotic *Lactobacillus acidophilus* strain INMIA 9602 Er 317/402 and putative probiotic lactobacilli on DNA damages in the small intestine of Wistar rats in *vivo* . Probiotics Antimicrob. Proteins 11, 905–909. doi: 10.1007/s12602-018-9491-y 30515721

[B66] Pepoyan A. Balayan M. Manvelyan A. Galstyan L. Pepoyan S. Petrosyan S.. (2018a). Probiotic *Lactobacillus acidophilus* Strain INMIA 9602 Er 317/402 administration reduces the numbers of *Candida albicans* and abundance of Enterobacteria in the gut microbiota of familial Mediterranean fever patients. Front. Immunol. 9. doi: 10.3389/fimmu.2018.01426 PMC6028570 29997616

[B67] Pepoyan A. Z. Balayan M. H. Manvelyan A. M. Mamikonyan V. Isajanyan M. Tsaturyan V. V.. (2017). *Lactobacillus acidophilus* INMIA 9602 Er-2 strain 317/402 probiotic regulates growth of commensal *Escherichia coli* in gut microbiota of familial Mediterranean fever disease subjects. Lett. Appl. Microbiol. 64, 254–260. doi: 10.1111/lam.12722 28140472

[B68] Pepoyan A. Balayan M. Manvelyan A. Pepoyan S. Malkhasyan L. Bezhanyan T.. (2018b). Radioprotective effects of lactobacilli with antagonistic activities against human pathogens. Biophys. J. 114, 665a. doi: 10.1016/j.bpj.2017.11.3586

[B69] Pepoyan A. Z. Balayan M. H. Manvelyan A. M. Tsaturyan V. V. (2014). Growth and motility of gut commensal *Escherichia coli* in health and disease. Biophys. J. 106, 726. doi: 10.1016/j.bpj.2013.11.4343

[B70] Pepoyan A. Z. Chikindas M. L. (2020). Plant-associated and soil microbiota composition as a novel criterion for the environmental risk assessment of genetically modified plants. GM Crops Food 11, 47–53. doi: 10.1080/21645698.2019.1703447 31847696 PMC7158920

[B71] Pepoyan A. Harutyunyan N. Grigoryan A. Balayan M. Tsaturyan V. Manvelyan A.. (2015). [The certain clinical characteristics of blood in patients with family Mediterranean fever disease of Armenian population]. Klin. Lab. Diag. 60, 46–47. 26466452

[B72] Pepoyan A. Z. Harutyunyan N. A. Pepoyan E. S. Tsaturyan V. V. Torok T. (2019b). Relationship between the numbers of *Candida albicans* and abundance of *Helicobacter* spp. in the gut microbiota of familial Mediterranean fever patients. Helicobacter 24, S1. doi: 10.1111/hel.12647

[B73] Pepoyan A. Z. Manvelyan A. M. Balayan M. H. McCabe G. Tsaturyan V. V. Melnikov V. G.. (2020a). The effectiveness of potential probiotics *Lactobacillus rhamnosus Vahe* and *Lactobacillus delbrueckii* IAHAHI in irradiated rats depends on the nutritional stage of the host. Probiotics Antimicrob. Proteins 12, 1439–1450. doi: 10.1007/s12602-020-09662-7 32462507

[B74] Pepoyan A. Z. Pepoyan E. S. Galstyan L. Harutyunyan N. A. Tsaturyan V. V. Torok T.. (2021). The effect of immunobiotic/psychobiotic *Lactobacillus acidophilus* strain INMIA 9602 Er 317/402 narine on gut prevotella in familial mediterranean fever: gender-associated effects. Probiotics Antimicro. Prot. 13, 1306–1315. doi: 10.1007/s12602-021-09779-3 34132998

[B75] Pepoyan A. Trchounian A. (2009). Biophysics, molecular and cellular biology of probiotic activity of bacteria,ed. by Trchunyan A.H. Research Signpost: Kerala, India. Bacterial Membr., 275–287.

[B76] Pepoyan A. Z. Tsaturyan V. V. Badalyan M. Weeks R. Kamiya S. (2020b). Chikindas, M.L. Blood protein polymorphisms and the gut bacteria: impact of probiotic *Lactobacillus acidophilus* Narine on *Salmonella* carriage in sheep. Benef Microbes 11, 183–189. doi: 10.3920/BM2019.0138 32028777

[B77] Pepoyan A. Tsaturyan V. Manukyan V. Egorov I. Ilina L. (2023). “Novel Probiotic *Lactiplantibacillus plantarum* str. ZPZ as a Possible Candidate for “One Health” Probiotic,” in Agriculture digitalization and organic production. Smart Innovation, Systems and Technologies. ADOP, vol. 362 . Eds. Ronzhin A. Kostyaev A. (Springer, Singapore). doi: 10.1007/978-981-99-4165-0_13

[B78] Pogany L. (2017). A placebo-hatás pszichobiológiai háttere és klinikai vonatkozásai a pszichiátriában [Psychobiological background and clinical aspects of the placebo effect in psychiatry]. Neuropsychopharmacol. Hung 19, 197–206. 29411707

[B79] Pronovost-Morgan C. Hartogsohn I. Ramaekers J. G. (2023). Harnessing placebo: Lessons from psychedelic science. J. Psychopharmacol. 37, 866–875. doi: 10.1177/02698811231182602 37392012 PMC10481630

[B80] Qin Y. Havulinna A. S. Liu Y. Jousilahti P. Ritchie S. C. Tokolyi A.. (2022). Combined effects of host genetics and diet on human gut microbiota and incident disease in a single population cohort. Nat. Genet. 54, 134–142. doi: 10.1038/s41588-021-00991-z 35115689 PMC9883041

[B81] Rahman M. Sabir A. A. Mukta J. A. Khan M. M. A. Mohi-Ud-Din M. Miah M. G.. (2018). Plant probiotic bacteria *Bacillus* and *Paraburkholderia* improve growth, yield and content of antioxidants in strawberry fruit. Sci. Rep. 8, 2504. doi: 10.1038/s41598-018-20235-1 29410436 PMC5802727

[B82] Rodriguez E. T. Flores H. E. M. Lopez J. O. R. Vega R. Z. Garciglia R. S. Sanchez R. E. P. (2017). Survival rate of Saccharomyces boulardii adapted to a functional freeze-dried yogurt: experimental study related to processing, storage and digestion by Wistar rats. Funct. Foods Health Dis. 7, 98–114. doi: 10.31989/ffhd.v7i2.319

[B83] Schaefer M. Kühnel A. Schweitzer F. Enge S. Gärtner M. (2023). Neural underpinnings of open-label placebo effects in emotional distress. Neuropsychopharmacology 48, 560–566. doi: 10.1038/s41386-022-01501-3 36456814 PMC9852452

[B84] Šefcová M. A. Ortega-Paredes D. Larrea-Álvarez C. M. Mina I. Guapás V. Ayala-Velasteguí D.. (2023). Effects of *Lactobacillus fermentum* administration on intestinal morphometry and antibody serum levels in *Salmonella*-Infantis-challenged chickens. Microorganisms 11, 256. doi: 10.3390/microorganisms11020256 36838221 PMC9963312

[B85] Seneviratne C. Noel J. Franklin P. D. Colloca L. (2022). Editorial: Harnessing placebo mechanisms. Front. Psychiatry 13. doi: 10.3389/fpsyt.2022.1022762 PMC9512394 36172509

[B86] Shafir R. Israel M. Colloca L. (2023). Harnessing the placebo effect to enhance emotion regulation effectiveness and choice. Sci. Rep. 13, 2373. doi: 10.1038/s41598-023-29045-6 36759537 PMC9911767

[B87] Shahinyan A. Garibyan J. Pepoyan A. Karapetyan O. (2003). Cancerolitic action of *E. coli* . J. Nat. Sci. 1, 53–58.

[B88] Shandilya S. Kumar S. Kumar Jha N. Kumar Kesari K. (2021). Ruokolainen, J.Interplay of gut microbiota and oxidative stress: Perspective on neurodegeneration and neuroprotection. J. Adv. Res. 38, 223–244. doi: 10.1016/j.jare.2021.09.005 35572407 PMC9091761

[B89] Singh S. Pal N. Shubham S. Sarma D. K. Verma V. Marotta F.. (2023). Polycystic ovary syndrome: etiology, current management, and future therapeutics. J. Clin. Med. 12, 1454. doi: 10.3390/jcm12041454 36835989 PMC9964744

[B90] Sonawalla S. B. Rosenbaum J. F. (2002). Placebo response in depression. Dialogues Clin. Neurosci. 4, 105–113. doi: 10.31887/DCNS.2002.4.1/ssonawalla 22034204 PMC3181672

[B91] Stepanyan K. Balayan M. H. Vassilian A. Pepoyan A. Z. Trchounian A. H. (2007). Growth peculiarities and some characteristics of membrane for probiotic strain of *Escherichia coli* . Memb. Cell Biol. 1, 333–335. doi: 10.1134/S1990747807040095.

[B92] Talerico R. Cardillo C. De Vito F. Schinzari F. Soldato M. Giustiniani M. C.. (2020). Mesothelioma in familial mediterranean fever with colchicine Intolerance: A case report and literature review. Front. Immunol. 11. doi: 10.3389/fimmu.2020.00889 PMC7237567 32477360

[B93] Touitou I. Pepoyan A. (2008). Concurrence of Crohn’s and familial Mediterranean fever diseases for Armenian cohort. Iflamm. Bowel. Dis. 14, S39. doi: 10.1097/00054725-200812001-00128

[B94] Tsaturyan V. Manvelyan A. Balayan M. Harutyunyan N. Pepoyan E. Torok T.. (2023). Host genetics and gut microbiota composition: Baseline gut microbiota composition as a possible prognostic factor for the severity of COVID-19 in patients with familial Mediterranean fever disease. Front. Microbiol. 14. doi: 10.3389/fmicb.2023.1107485 PMC10098164 37065143

[B95] Tsaturyan V. Poghosyan A. Toczyłowski M. Pepoyan A. (2022). Evaluation of malondialdehyde levels, oxidative stress and host-bacteria interactions: *Escherichia coli* and *Salmonella* derby. Cells 26;11, 2989. doi: 10.3390/cells11192989 PMC9564265 36230950

[B96] van Leeuwen P. T. Brul S. Zhang J. Wortel M. T. (2023). Synthetic microbial communities (SynComs) of the human gut: design, assembly, and applications. FEMS Microbiol. Rev. 47, fuad012. doi: 10.1093/femsre/fuad012 36931888 PMC10062696

[B97] Wan X. Yang Q. Wang X. Bai Y. Liu Z. (2023). Isolation and cultivation of human gut microorganisms: A review. Microorganisms 11, 1080. doi: 10.3390/microorganisms11041080 37110502 PMC10141110

[B98] Wang L. Sun H. Gao H. Xia Y. Zan L. Zhao C. A. (2023). meta-analysis on the effects of probiotics on the performance of pre-weaning dairy calves. J. Anim. Sci. Biotechnol. 14, 3. doi: 10.1186/s40104-022-00806-z 36597147 PMC9811714

[B99] Welzel T. Benseler S. M. Kuemmerle-Deschner J. B. (2021). Management of monogenic IL-1 mediated autoinflammatory diseases in childhood. Front. Immunol. 12. doi: 10.3389/fimmu.2021.516427 PMC8044959 33868220

[B100] Yetman H. E. Cox N. Adler S. R. Hall K. T. Stone V. E. (2021). What do placebo and nocebo effects have to do with health equity? the hidden toll of nocebo effects on racial and ethnic minority patients in clinical care. Front. Psychol. 12. doi: 10.3389/fpsyg.2021.788230 PMC8733207 35002881

[B101] Zadeh N. Getzug T. Grody W. (2011). Diagnosis and management of Familial Mediterranean Fever: Integrating medical genetics in a dedicated interdisciplinary clinic. Genet. Med. 13, 263–269. doi: 10.1097/GIM.0b013e31820e27b1 21317656

[B102] Zengler K. Hofmockel K. Baliga N. S. Behie S. W. Bernstein H. C. Brown J. B.. (2019). EcoFABs: advancing microbiome science through standardized fabricated ecosystems. Nat. Methods 16, 567–571. doi: 10.1038/s41592-019-0465-0 31227812 PMC6733021

[B103] Zheng D. Liwinski T. Elinav E. (2020). Interaction between microbiota and immunity in health and disease. Cell Res. 30, 492–506. doi: 10.1038/s41422-020-0332-7 32433595 PMC7264227

